# Targeting focal adhesion kinase reprograms the pancreatic tumor microenvironment and renders pancreas cancer responsive to checkpoint immunotherapy

**DOI:** 10.1186/2051-1426-3-S2-P400

**Published:** 2015-11-04

**Authors:** Hong Jiang, Brett Knolhoff, Yu Zhu, John Herndon, Irina Shapiro, David Weaver, Jonathan Pachter, Andrea Wang-Gillam, David G DeNardo

**Affiliations:** 1 Washington University St. Louis, School of Medicine, St. Louis, MO, USA; 2 Verastem Inc., Needham, MA, USA

## 

Checkpoint immunotherapeutics are promising agents with potential to improve patient outcomes in several cancer types. Unfortunately, to date, single agent immunotherapy has achieved limited clinical benefit in patients with pancreatic ductal adenocarcinoma (PDAC). This may be due to the presence of the uniquely immunosuppressive tumor microenvironment present in PDACs that creates a barrier to immune surveillance by T cells. Critical obstacles to immunotherapy in PDAC tumors include the dense desmoplastic stroma that acts as a barrier to T cell infiltration and high numbers of tumor-associated immunosuppressive cells, such as MDSCs and regulatory T cells (Tregs).

To understand which signaling pathways in pancreatic tumor cells might drive this suppressive tumor microenvironment, we analyzed the correlation between hyper-activated signaling molecules and tumor infiltrating leukocytes using 50 human PDAC tumor tissues. Of the pathways evaluated, we found that focal adhesion kinase (FAK) activity is elevated in human PDAC and that FAK activity correlates with highly fibrotic tumors with poor CD8^+^ T cell infiltration. The oral FAK kinase inhibitor VS-4718, currently in Phase I clinical evaluation, was tested to determine if it could overcome the immunosuppressive tumor microenvironment of PDAC. Single agent VS-4718 dramatically limited tumor progression resulting in a doubling of survival in the p48-CRE/Kras^G12D^/p53^flox/+^ PDAC mouse model (KPC mice). This alteration in tumor progression was associated with dramatically reduced tumor fibrosis, decreased numbers of FOXP3^+^ Tregs and tumor-infiltrating myeloid cells, and anti-tumor polarization of tumor-associated macrophages.

We postulated that the desirable effects of FAK inhibition on the tumor microenvironment might render PDAC tumors more sensitive to immunotherapy. Accordingly, we found that VS-4718 significantly potentiated the efficacy of anti-PD-1 and anti-CTLA4 antibodies in KPC mouse models nearly tripling survival times. We next assessed the mechanism of this potentiation. We found that FAK in tumor cells regulates pro-inflammatory and pro-fibrotic cytokine secretion. Furthermore, we found that shRNA knockdown of FAK in PDAC cells results in failure of PDAC cells to induce pro-fibrotic programs in fibroblasts. Importantly, the FAK inhibitor VS-4718 and FAK shRNA in the tumor cells were each effective in increasing CD8+ cytotoxic T cell infiltration into the PDAC tumors *in vivo*. Taken together, these data suggest that FAK inhibition increases immune surveillance programs in PDAC tumors by overcoming the fibrotic and inflammatory microenvironment rendering tumors more responsive to immunotherapy. These data provide rationale for clinical evaluation of a FAK inhibitor in combination with a PD-1 or PD-L1 antibody in patients with pancreatic and other cancers.

